# Is prolactin receptor signaling a target in dopamine-resistant prolactinomas?

**DOI:** 10.3389/fendo.2022.1057749

**Published:** 2023-01-12

**Authors:** Jimena Ferraris

**Affiliations:** Department of Biophysics and Biochemistry, Stockholm University, Stockholm, Sweden

**Keywords:** prolactinomas, PRL, PiNETs, PRL receptor, JAK/STAT, PI3K/AKT

## Abstract

The hypothalamic neuroendocrine catecholamine dopamine regulates the lactotroph function, including prolactin (PRL) secretion, proliferation, and apoptosis. The treatment of PRL-secreting tumors, formerly known as prolactinomas, has relied mainly on this physiological characteristic, making dopamine agonists the first therapeutic alternative. Nevertheless, the group of patients that do not respond to this treatment has few therapeutical options. Prolactin is another physiological regulator of lactotroph function, acting as an autocrine/paracrine factor that controls PRL secretion and cellular turnover, inducing apoptosis and decreasing proliferation. Furthermore, the signaling pathways related to these effects, mainly JAK/STAT and PI3K/Akt, and MAPK, have been extensively studied in prolactinomas and other tumors as therapeutic targets. In the present work, the relationship between PRL pathophysiology and prolactinoma development is explored, aiming to comprehend the value of PRL and PRLR-associated pathways as exploratory fields alternative to dopamine-related approaches, which are worth physiological characteristics that might be impaired and can be potentially restored or upregulated to provide more options to the patients.

## 1 Introduction

Pituitary neuroendocrine tumors (PitNETs), formerly pituitary adenomas, are systematized according to the 2022 WHO classification, accounting for the expression of transcription factors, hormones, and biomarkers. Lactotroph tumors (commonly referred to as prolactinomas) are a type of Pit-1-linage PitNET characterized by the presence of PRL, either in paranuclear dot-like expression (“Sparsely granulated lactotroph tumor”) or a diffuse cytoplasmatic manner (“Densely granulated lactotroph tumor”). Other PitNET-expressing PRL includes the Mammosomatotroph tumor, the Mature plurihormonal PIT1-lineage, the Immature PIT1-lineage tumor, and the Acidophil stem cell tumor and Mixed somatotroph and lactotroph tumor (1).

The present review will discuss the relationship between PRL and the pathogenesis of PRL-related neuroendocrine tumors, focusing primarily on non-aggressive lactotroph tumors, aiming to identify targets for upcoming treatment strategies.

Lactotroph PiNETs are benign adenomas and constitute about 50% of pituitary tumors, with a prevalence that ranges from 25 to 63/100000, depending on the region reported (2), and an annual incidence of 4 new cases per 100,000 inhabitants (3).

The clinical consequences of lactotroph adenomas are concomitant hyperprolactinemia and mechanical compression effects at the brain level exerted by the presence of the tumor. These aspects have been previously reviewed, and the reader can refer to Melmed et al. (5)or Karavitaki (3) for further details.

The first-line treatment, dopamine receptor 2 (D2R) agonist administration, reduces PRL levels and tumor size. The Endocrine Society recommends administering cabergoline to treat hyperprolactinemia in patients presenting macroadenomas (4). However, 20%-30% of patients do not respond to treatment (2, 4–6).

The pathogenesis of PRL-secreting PiNETs has been extensively investigated. Two germline mutations induce familial prolactinomas: MEN1 and AIP mutations (5). However, spontaneous pituitary adenomas are the most prevalent, and these tumors’ pathophysiology remains elusive. Being dopamine a natural inhibitor of PRL secretion and lactotrophproliferation; and the dopamine pathway a successful target, most efforts have been made to understand the pathophysiology of the dopamine and dopamine-associated pathways, such as the biology of dopamine receptors, extracellular-regulated mediators such as TGF-beta or intracellular signaling pathways such as ERK1/2 (7–9).

Nevertheless, in this interconnected network of neuroendocrine, endocrine, and local factors crosstalk, PRL is the precise outcome, but that could also be an initiator or intermediate player. So, this review will summarise the current knowledge about the relationship between PRL and pituitary physiology and aim to identify PRL’s role in the pathophysiology of prolactinomas. Is PRL a pro or anti-prolactinoma factor?

## 2 Prolactin and prolactinomas: A retrospective viewpoint

Hypothalamic neurons of the tuberoinfundibular (TIDA) and tuberohypophysial dopamine systems express PRL receptors (PRLR), so they are sensitive to changes in circulating levels of this hormone. Circulating PRL reaches the arcuate nucleus and stimulates the synthesis and activity of the tyrosine hydroxylase in the TIDA neurons, which increases dopamine release to the portal system, inhibiting the secretory activity of lactotrophs. This way, PRL regulates its synthesis and release by controlling hypothalamic dopamine secretion.

At the pituitary level, PRL can induce a negative feedback control strategy. Prolactin inhibits its production and secretion (10), and as it will be discussed later, it inhibits cellular proliferation. Notably, this effect contrasts PRL in many other target tissues, such as the mammary gland, lymphoid cells, pancreas, or the prostate, where the main physiological action of PRL is pro-proliferative. Prolactin has been implicated in tumorigenesis in some tissues, like the mammary gland and prostate (reviewed in (11)), and others, such as glioblastomas.

So, in the context of prolactinomas, what has sounded intuitively comprehensive has been that PRL, being elevated in the pathological context, could be contributing to prolactinoma proliferation and creating a positive loop: high PRL levels lead to enhanced lactotroph activity and, thus, contributes to, at least, the prolactinoma progression.

Early studies proposed PRL as a growth factor in a somatotroph-derived cell line, GH3 (12). Later, it was demonstrated that PRL inhibits its transcription, controlling its production through an ultra-short feedback loop (10). Dopamine receptor 2 KO mice (D2RKO) develop pituitary hyperplasia and hyperprolactinemia. Consequently, it was proposed that those tumors were consequences of the increased levels of PRL in these animals, assigning PRL a proliferative action on pituitary cells, especially lactotrophs (13).

On the other hand, PRL Receptor KO mice (PRLRKO) present hyperprolactinemia and develop prolactinomas after 12 months of age with high penetrance (6). However, the seminal work by Schuff et al. showed that *in vivo*, constitutive double D2RKO/PRLRKO mice also exhibit prolactinomas, even significantly higher than single knockouts. This observation led to questions about whether there are independent actions of PRL on lactotroph cells (13).

The same group explored the effects of PRL in cultured lactotroph cells from wild-type and D2RKO mice, as they hypothesized a dopamine-independent PRL effect. They observed that PRL treatment reduces the proliferative index of lactotroph proliferation from wild-type female animals, whereas PRL has little effect in cultured lactotrophs derived from hyperprolactinemic D2RKO animals. Another exciting aspect is that although cabergoline restores circulating PRL levels in PRLRKO mice, it does not induce tumor reduction, suggesting that dopamine and PRL effects can be interplaying but also have separate actions (6).

Many years later, conditional deleting of the PRLR, specifically in lactotrophs, showed no effect on PRL levels, and the authors did not observe changes in pituitary size. The deletion was achieved in 20% of pituitary cells leading to a qualitative reduction in one of the PRLR-mediated signaling activation, pSTAT5. Interestingly, these mice presented an elevated dopamine tone, suggesting a strengthening in the inhibitory input as a compensatory mechanism of the constitutive deficiency of PRLR inhibitory effect in lactotrophs (14).

So far, all these backgrounds suggested that 1) PRL can exert an effect on lactotrophs inhibiting proliferation, 2) That effect is independent of dopamine, and 3) In a hyperprolactinemic context, this physiological mechanism could be impaired.

Apart from the knockout mouse models described above, other evidence suggested that PRL could be implicated in regulating lactotroph cell turnover. In rats, two-week treatment with estradiol leads to hyperprolactinemia. Although pituitary hyperplasia is observed in this animal model, the apoptotic rate of hyperprolactinemic estradiol-treated rats is higher than control ovariectomized females (15). Although the role of dopamine and estrogens themselves could not be excluded at the time, the presence of PRL and an elevated apoptotic rate was suggestive of a relationship between PRL and the regulation of pituitary turnover.

Nevertheless, a question remained elusive: Does PRL act directly on lactotrophs through PRLR activation?

## 3 Prolactin effects on pituitary lactotrophs: Evidence for direct effects

Apart from the knockout mouse model and the chronic estradiol treatment described above, other evidence suggests that PRL can be implicated in regulating lactotroph cell turnover *in vivo*. One is that the induction of acute hyperprolactinemia by PRL injection leads to a decrease in pituitary proliferation and an increase in the apoptotic rate, particularly in lactotroph cells. The same is observed when hyperprolactinemia is induced by acute treatment with a D2R antagonist. This evidence illustrates a possible dopamine-independent effect of PRL on lactotrophs (16).

The implication of a PRLR-mediated effect of PRL was further confirmed in male and female transgenic mice constitutively expressing a PRLR antagonist. Both males and females that lack PRLR activation either by the presence of a PRLR antagonist or by lacking PRLR (e.g., PRLR KO mice) present pituitary hyperplasia and altered proliferation and apoptotic rates (16, 17).

Interestingly, circulating hormones regulate anterior pituitary cell proliferation and apoptotic rates in female rodents. The proestrus seems to be an essential regulation point of cellular homeostasis at the pituitary level. Estradiol, TNF-Alpha, FasL, and dopamine induce apoptosis, particularly during this estrous cycle stage. The highest proliferative rate occurs in estrus, whereas the highest apoptotic rate occurs in proestrus, leading to a balance in the apoptosis/proliferation rate in the tissue. This apoptosis peak coincides with the PRL peak and is absent in PRLRKO females, even before tumor formation (around 6 months old), although hyperprolactinemia has been evident since early ages (6, 13). Thus, a cumulative lack of PRLR-dependent apoptosis could explain the later pituitary hyperplasia in this animal model (16).

The alteration of low but recurrent apoptotic rates was also observed in females where the PRLR was constitutively antagonized. These mice also present an altered proliferation rate and develop pituitary hyperplasia (16).

Studying autocrine factors can be challenging since adding the agonist to a system already exposed to that factor can mask some effects, pushing the system to non-physiological conditions. So, it was not until later, with the use of a PRLR antagonist, that question could be further clarified (18).

The inhibition of the PRLR activation by locally produced PRL showed that local PRL acts as a proapoptotic and antiproliferative factor in both primary cultures and the tumor-derived GH3 cell line (16, 17).

This body of evidence supports the physiological Role of autocrine/paracrine PRL in modulating cell turnover homeostasis and that alterations in this mechanism could lead to enhanced pituitary tumorigenesis.

## 4 Mechanism of action of PRL in Lactotrophs

PRL acts through a receptor belonging to the class I cytokine receptor group, a group of transmembrane-step proteins that share conserved sites in the extracellular and intracellular domain and do not possess intrinsic tyrosine kinase activity (19). Alternative processing of the primary transcript of the PRLR gene gives rise to different isoforms, which differ in the length of the amino acid chain of the intracellular portion but share identical extracellular portions and transmembrane domains (19–21). These isoforms are called long and short (or several types of short isoforms depending on the species) because of the length of their intracellular portion (358 and 57 amino acids, respectively) (19). The long isoform contains the *box* 1 and 2 regions, while the short isoforms lack the latter (22, 23).

The phosphorylation of PRLR depends on the binding of the intracellular portion of PRLR to intracytoplasmic kinases. PRLR is constitutively associated with proteins in the Janus kinase family, specifically, the JAK2 protein. Phosphorylated tyrosine residues possess the ability to bind transcription factors with SH2 domains, such as the family of transducer and transcription activator proteins (STAT, *signal transducer and activator of transcription*). After being phosphorylated, STAT proteins translocated to the nucleus and modulate the expression of specific genes (11, 20). The STAT family of proteins includes STAT 1, 3, and 5, and the latter is most often associated with the PRLR signaling pathway (18, 24). While all class I cytokine receptors can recruit proteins from the STAT family, the specificity of signaling occurring by binding a specific ligand to a given receptor is given by the subset of STAT proteins that each receptor recruits. Thus, it has been postulated that signaling through JAK2/STAT5 would be the specific pathway of the PRLR (24). Other proteins with the SH2 domain can be recruited by PRLR, such as the socs family proteins, SOC1-SOC7, and CIS (20). These PRL-induced proteins bind to and inhibit JAK2 activity by forming JAK-SOCS or JAK-SOCS-PRLR complexes. In addition, PRL induces the expression of the protein inactivator of *activated STAT* (PIAS). These proteins exert negative feedback by inhibiting the JAK/STAT signaling pathway, inhibiting PRL signaling. In addition to the JAK/STAT pathway, PRLR is very well known to activate other signaling pathways such as MAPK, Src (21), phosphoinositide-3 Kinase (PI3K)/Akt (25), or Nek3-vav2-Rac1 (22).

Since the JAK2 protein is associated with the intracellular portion proximal to the membrane, both LPRLR and SPRLR can bind to this enzyme. However, only the long isoform is phosphorylated by the activation of JAK2 since the tyrosine residues of the receptor susceptible to being phosphorylated in the terminal C portion of the PRLR are not present in the short isoform of the receptor (20). Therefore, PRL can activate or inhibit other pathways, such as MAPK and phosphatidylinositol 3 kinase (PI3K), without recruiting STAT proteins (21, 26, 27). In breast cancer cell-derived cell lines, PRL activates both Src family kinases and the JAK/STAT, as well as PI3K/Akt and MAPK signaling pathways. Whereas activation of MAPK occurs independently of STATs protein recruitment, it depends on JAK activation with PI3K as an intermediate cascade (26). In the ovary, PRL activates ERK1/2 and p38 MAPK independently of the JAK/STAT pathway by specific activation of the short isoform of the receptor (28). Hepatocytes express the PRLR short isoform in rodents (29, 30), and PRL inhibits the MAP3K-/c-Myc pathways in these cells Since the PRL action is mediated by that isoform of the PRLR (31), whereas other actions are mediated by the PRLR Long/JAK/STAT5 pathways (32, 33).

Adding to the complexity of the PRL/PRLR isoform and signaling puzzle, the expression of PRLR can be modulated by endocrine factors. Apart from sex differences, in hormone-responsive tissues, the expression of PRLR is variable in either reproductive stages or along the sexual cycle (16, 24, 28, 29, 34–36).

The rat, mouse and human adenohypophysis express both isoforms of PRLR (16, 29, 37–39). While the ratio of LPRLR to SPRLR isoforms is approximately 13:1 in males, it is variable along the estrous cycle in females, and the PRLR expression is higher in diestrus, with changes in the ratio that varies from approximately 36:1 in diestrus to 1:1 in proestrus (16, 17, 29).

Since both LPRLR and SPRLR isoforms are expressed in the pituitary, either isoform could mediate the effect of PRL action in lactotrophs. In this regard, a study showed that mice lacking the LPRLR isoform present high serum prolactin levels. This indicates a partial impairment in the negative feedback mechanism acting in the hypothalamus and the pituitary, supporting a role for the long isoform of the PRLR in controlling PRL levels (22).

## 5 Prolactin, prolactin receptor, and signaling pathways associated with the control of cellular turnover

The lactotroph function is controlled by several intracellular pathways controlling hormone production, secretion, and cell survival.

Prolactin gene expression is modulated by various signals, stimulatory such as estradiol and inhibitory such as dopamine, that Converge in several signaling pathways such as the AMPc/PKA, PKC, or MAPK pathways (19, 40, 41). The secretion of PRL is another control point, regulated mainly through calcium-dependent- mechanisms (42, 43) which can depend on the cell’s electrical activity, e.g., voltage-dependent calcium entry or signaling molecules such as IP3, initiated chiefly by G_q/11_-coupled membrane receptors (44).

The specific intracellular signals that control lactotrophs’ proliferation, death, and phenotype under physiological and pathological conditions also result from systemic, hypothalamic, and intrahypophyseal signals. Regardless of the signal trigger (estrogens (45–47), dopamine (8, 48), or TGF-β (9), for example), some intracellular signaling pathways have been identified as critical regulators of proliferation and apoptosis in both normal and tumoral lactotrophs. All these pathways are also susceptible to modulation by PRL.

The MAPK pathway is a pathway in which several extracellular signals converge, and particularly ERK is dysregulated in cell lines derived from prolactinomas (49, 50). The PI3K-Akt pathway is a proliferative pathway inhibited by dopamine, which also regulates the MAPK/ERK pathway, and both pathways work together, regulating cell proliferation (51). However, a Ras/MAPK mutation alone does not promote tumorigenesis in lactotroph cells (7). TGF-beta regulates transcription by recruitment of Smad proteins but also, through its so-called non-canonical pathway, regulates ERK1/2 and Jun kinases, PI3K, and Akt proteins (52, 53).

A balance between proliferation and apoptosis keeps the cell turnover. The evidence of factors controlling lactotrophs apoptosis has been less studied than the proliferative factors. Dopamine and estradiol have been extensively studied among the apoptosis factors for lactotroph cells. It was described that dopamine induces adenohypophysis cell apoptosis by activating p38 MAPK or oxygen-reactive species generated by dopamine metabolism (48, 54), by activation of the MEK/ERK1/2 pathways (55), and estrogens sensitize to cytokine-induced cell death by regulating transcription factor NFК-B (56) and protein balance of the Bcl-2 family (57). This apoptotic protein family is modulated by dopamine (58) and PRL.

The activation of PRLR leads to the phosphorylation of JAK and nuclear translocation of phosphorylated STAT5. Although PRLR-activated pathways are usually associated with cell differentiation or proliferative effects (11, 19–22), these pathways can also induce apoptotic effects. For example, STAT5 phosphorylation mediates the apoptosis of osteosarcoma-derived cells and cerebellar neurons by regulating the Bax/Bcl-2 ratio (59–61). The JAK2/STAT5-dependent balance towards proapoptotic Bax proteins leads to apoptosis in lactotroph cells (62).

PRLR downregulates MEK/Erk1/2 and PI3K/Akt pathways, leading to apoptosis and decreased proliferation (62). Furthermore, the mutation of a splicing factor, SF3B1, was associated with a bad prognosis. This mutation stimulates the PI3K/Akt pathway in prolactinomas, increasing tumor invasiveness (63). Similar pathways have been identified as therapeutic targets in prolactinoma by studying differentially expressed mRNA together with microRNAs (64).

In their recent review, Biagetti et al. identified potential therapeutic options related to relevant signaling pathways for the treatment of dopamine-resistant prolactinomas, highlighting the JAK/STAT3, PI3K-Akt-mTOR, MAPK/AMPK, and JAK2/STAT5 pathways. All of them are related to paracrine/autocrine PRL effects in the pituitary; for all, there are already described pharmacological modulators and thus are relevant pharmacological targets for potential aggressive prolactinomas. Nevertheless, no clinical trial currently assesses these therapeutic options (65).

## 6 Prolactin receptor expression and associated genetic alterations related to PRL-secreting adenomas

Suppose the PRLR mediates a physiological autocrine/paracrine control of the lactotroph population by PRL. In that case, mutations in this receptor are expected to be related to the formation, progression, or prognosis of PRL-secreting adenomas.

In 2013, a loss-of-function PRLR mutation was described in the extracellular domain-encoding region. The mutation was present in a family with autosomal dominant hyperprolactinemia. This mutation leads to an impairment in the JAK2/STAT5 signaling, and although no changes in the pituitary size were observed at the time of the study, this can indicate that the PRLR/JAK2/STAT5 activation can be a relevant control mechanism of lactotroph function in humans (66).

The first analysis of inactivating germline mutations of PRLR was not associated with prolactinomas concluding that most prolactinomas occur independently of germline changes in the PRLR gene (67). Nevertheless, in 2019, two germline PRLR intracellular domain variants were later associated with prolactinoma manifestation. Interestingly, one of those variants results in the overactivation of the Akt-related pathways (68).

Although genetic mutations are not the leading cause of prolactinoma development, since PitNETs are mainly sporadic (4, 69), the studies mentioned above can shed light on the mechanisms that could be altered during the initial phases of prolactinoma development.

Since both loss-of-function and gain-of-function genetic alterations can lead to alteration in the lactotroph function, it is possible that a balance between PRLR cascades plays a role in the maintenance of lactotrophs homeostasis and that the lack of equilibrium in the intricated pathway network, as discussed previously, can lead to clinical manifestations. Given the complexity of the PRL and lactotroph turnover regulation, more efforts should be put into understanding the interconnections between receptors, isoforms, and signaling pathways to elucidate the physiological relevance of PRLR in the control of lactotroph function *in vivo*.

## 7 Discussion

Prolactin-secreting PiNETs that do not respond to standard treatments with dopamine agonists imply a large number of patients annually around the globe. It has been proposed that prolactinomas have a monoclonal origin (4), and although several oncogenes are overexpressed in these tumors, the pathophysiological processes that lead to the formation of prolactinomas have not yet been established (4, 7, 19). From the analysis of familial pituitary tumors, a series of oncogenes involved in tumor development have been proposed, but most prolactinomas (more than 95%) occur spontaneously, and these oncogenes do not explain their appearance (7, 70). Although progression to invasive and metastatic tumors is rare, lactotroph macroadenomas are one of the predominating types (71–73), and the mechanism leading to malignant transformation is currently unknown (74).

Since the adenohypophysis is a gland with high plasticity (75), alterations in the mechanisms that normally regulate adenohypophysis cell renewal could be involved in developing pituitary tumors (38).

The evidence presented here suggests a significant role of PRL in the pathogenesis of prolactinomas. Such implications can be considered in two main scenarios. In one scenario, alteration of PRLR-related actions locally at the pituitary level, either initiating or contributing to tumor development. The second is the effect of PRL at the hypothalamic level, controlling neuroendocrine functions, such as dopamine or potentially other hypothalamic factors, that further control the pituitary’s cell physiology.

At the hypothalamic level, prolactin feedback onto TIDA neurons contribute to maintaining lactotroph homeostasis by negative feedback that restores dopamine inhibitory input to the pituitary (76). In the adenohypophysis, PRL possesses proapoptotic and anti-proliferative effects, which are critical for maintaining tissue homeostasis of the gland in rodent models, in an interplay with mainly hypothalamic factors (13, 14, 16, 17, 77). Deficiencies in PRLR signaling due to PRLR activity alterations or wrong intracellular pathway connectivity, crosstalk, or co-regulation exerted by other factors, such as hypothalamic or paracrine mechanisms, can lead to pituitary hyperplasia and eventual tumor development.

The intracellular signals that regulate the specific phenotype of lactotrophs, as well as the control of their proliferation and the death of these cells, are very little known in humans (7, 78). Approaching how prolactinomas develop from studying intracellular signaling pathways that regulate the proliferation and apoptosis of lactotrophs and the study of a physiological regulator of these pathways, PRL, is necessary to understand the pathophysiology of the development of tumors in this gland. Identifying therapeutic targets that contribute to the design of new treatments will be possible if new hypotheses are tested and efforts are currently required to understand the mechanisms in human pituitaries.

Prolactin, dopamine and other factors control lactotroph homeostasis (7, 19, 48, 52, 65). For patients where dopamine agonists are inefficient, it is worth consideringwhether the pathogenesis of those tumors is the same as in those responsive to dopamine. The field usually includes prolactinomas in a unique group in which, first, a dopamine agonist is administered, and in case of treatment failure, surgery and a very limited pharmacological toolbox are considered, although the probability of success is decreased (2, 79). Merely adding other players in the lactotroph physiological regulation may help to understand if tumors categorized as “refractory to treatment with dopamine agonists” involve a different pathophysiological mechanism.

If such factors can be identified, the exploration, for example, of PRLR or PRLR-associated pathways, not only in terms of mutations but also in gene expression regulation or modulatory molecules using high throughput technologies in patients, could help in designing a specific personalized therapy (63, 65, 67, 68),

The approach to the knowledge of how prolactinomas develop from studying physiological factors that control the intracellular signaling pathways that regulate the proliferation and apoptosis of lactotrophs is critical, and PRL is a promising candidate.

## Author contributions

The author confirms being the sole contributor of this work and has approved it for publication.
